# The *Pseudomonas aeruginosa* Transcriptome in Planktonic Cultures and Static Biofilms Using RNA Sequencing

**DOI:** 10.1371/journal.pone.0031092

**Published:** 2012-02-03

**Authors:** Andreas Dötsch, Denitsa Eckweiler, Monika Schniederjans, Ariane Zimmermann, Vanessa Jensen, Maren Scharfe, Robert Geffers, Susanne Häussler

**Affiliations:** 1 Chronic Pseudomonas Infections, Helmholtz Center for Infection Research, Braunschweig, Germany; 2 Genome Analytics, Helmholtz Center for Infection Research, Braunschweig, Germany; 3 Pathophysiology of Bacterial Biofilms, Twincore, Center for Clinical and Experimental Infection Research, joint venture of the Helmholtz Center for Infection Research and the Medical School Hannover, Hannover, Germany; Niels Bohr Institute, Denmark

## Abstract

In this study, we evaluated how gene expression differs in mature *Pseudomonas aeruginosa* biofilms as opposed to planktonic cells by the use of RNA sequencing technology that gives rise to both quantitative and qualitative information on the transcriptome. Although a large proportion of genes were consistently regulated in both the stationary phase and biofilm cultures as opposed to the late exponential growth phase cultures, the global biofilm gene expression pattern was clearly distinct indicating that biofilms are not just surface attached cells in stationary phase. A large amount of the genes found to be biofilm specific were involved in adaptation to microaerophilic growth conditions, repression of type three secretion and production of extracellular matrix components. Additionally, we found many small RNAs to be differentially regulated most of them similarly in stationary phase cultures and biofilms. A qualitative analysis of the RNA-seq data revealed more than 3000 putative transcriptional start sites (TSS). By the use of rapid amplification of cDNA ends (5′-RACE) we confirmed the presence of three different TSS associated with the *pqsABCDE* operon, two in the promoter of *pqsA* and one upstream of the second gene, *pqsB*. Taken together, this study reports the first transcriptome study on *P. aeruginosa* that employs RNA sequencing technology and provides insights into the quantitative and qualitative transcriptome including the expression of small RNAs in *P. aeruginosa* biofilms.

## Introduction

The study of gene expression as a function of its adaptation to a specific niche reveals important insights into how bacteria sense and respond to varied habitats. Thereby, the question of how gene expression differs in mature biofilms as opposed to planktonic cells has become a major research focus in the last decade [1]–[4], because the biofilm mode of growth is intimately connected to chronic persistent infections [5]–[7]. Chronic infections are extremely difficult to treat: biofilm bacteria do not only withstand common antimicrobial therapy but the structured community of cells embedded in an extracellular polymeric (EPS) matrix is also efficiently protected from the immune defenses of the host [8]–[10]. One example of a chronic infection of major clinical importance is the persistent colonization of the lungs of cystic fibrosis patients with *Pseudomonas aeruginosa* [11], [12]. Once established, these chronic infections cannot be eradicated despite even intensified antimicrobial therapy [8], [12], [13].

Biofilm studies are extremely sensitive to strain and experimental differences [14], nevertheless several studies on gene expression in *P. aeruginosa* biofilms have revealed distinct transcriptional responsive patterns to the biofilm niche [1], [4], [15]–[22]. We have recently presented a method for the analysis of biofilm growth based on automated microscopy in a 96-well plate system [23], which has already been used for a global screen for genetic determinants of biofilm formation in *P. aeruginosa* [24]. The static growth conditions in this setup differ from previous studies that applied variations of a continuous flow setup, where the biofilms grow under a steady supply of fresh nutrients. Furthermore, whereas the previous transcriptome analyses have relied on hybridization-based methods where cDNA was hybridized to microarray chips, today second generation sequencing technology has the potential to improve functional genomics experiments, including quantitative gene expression studies at a much greater dynamic range for measuring variability in expression levels [25], [26]. In addition, the commercially available and widely used PaeG1a microarray (Affymetrix) is almost entirely restricted to genes that are present in the PAO1 genome and also lacks non-coding genes especially those of small regulatory RNAs (sRNAs), a limitation that can also be overcome by sequencing based transcriptome analysis.

In this study, we analyzed the global gene expression of *P. aeruginosa* biofilms growing in the static 96-well setup that was recently introduced as a high-throughput system for analysis of biofilm formation [23], [24]. A comparative analysis of RNA seq data of late exponential and stationary planktonic and biofilm cultures of the widely used strain PA14 revealed a set of genes under biofilm-specific regulation that largely compares with previously published biofilm specific genes and uncovered differential expression of small RNAs in *P. aeruginosa* stationary phase and biofilm cultures.

## Results and Discussion

### Sequencing of the *P. aeruginosa* PA14 transcriptome

In this study we aimed at using RNA sequencing for the identification of the global gene expression pattern in *P. aeruginosa* when grown within biofilms. Since we have recently used a 96-well plate format static biofilm assay to screen the Harvard Medical School PA14 transposon mutant library [27] for mutants that are affected in biofilm formation [24], we decided to also use the *P. aeruginosa* PA14 strain in this study. PA14 forms well-structured biofilms in the 96-well plates despite the fact that PA14 is missing a functional LadS protein, which was shown to be involved in the regulation of biofilm formation [28]. Total RNA was isolated from two biological replicates of planktonic cultures of *P. aeruginosa* PA14 in the late exponential (P-4) and stationary growth phase (P-12) as well as from static biofilm cultures that were harvested after 24 h (B-24) and 48 h (B-48), respectively. After depletion of rRNA by the use of a commercial capture and depletion system, strand-specific barcoded cDNA libraries were generated from the RNA-samples and all samples were sequenced on two lanes of an Illumina GenomeAnalyzer-IIx. The raw sequence output consisted of 84.1 million reads, each with a length of 36 nucleotides. Alignments to the PA14 genome were generated and non-ribosomal reads that did not align uniquely with the genomic sequence were discarded. Although an rRNA depletion step based on specific oligonucleotide capture of the 16 S and 23 S subunits (MICROBExpress Kit by Ambion, see Material & Methods section for details) was included and the final libraries were normalized using double-strand specific nuclease (DSN), the fraction of mappable non-ribosomal cDNA reads was very low for the biofilm samples (approx. 5% as compared to up to 30% for exponential phase cultures). A more efficient removal of ribosomal RNA sequences has been reported for *E. coli* (up to 60%) using either of the methods that were applied here [29]. Obviously the efficiency of rRNA removal strongly depends on the culture conditions, as lower proportions of mRNA reads were consistently sequenced from biofilm cultures that might have higher rRNA:mRNA ratios than those of planktonic cultures.

**Figure 1:**
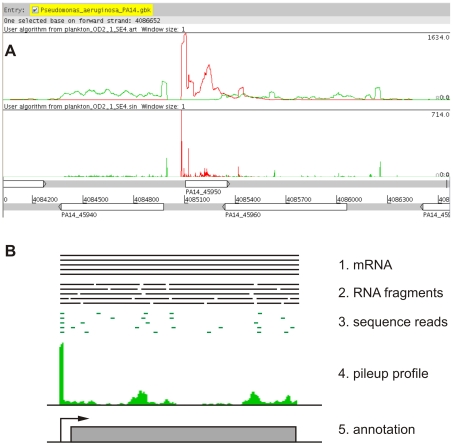
Profiles displaying the genome coverage by sequence reads. A) Profiles showing the coverage of the plus (red) and minus (green) strand of the *P. aeruginosa* PA14 genome visualized using the Artemis genome browser [58]. The upper profile represents a *pileup* of the sequence reads counting the number of reads overlapping each position of the chromosome. In the lower *sinister* profile, only left end (5′-) positions of sequence reads were counted. The genome annotation is shown below the profiles with numbers indicating the position on the chromosome and white boxes indicating genes on the plus strand (upper row) and the minus strand (lower row), respectively. The genes within the displayed region are *lasI* (PA14_45940), *rsaL* (PA14_45950) and *lasR* (PA14_45960). B) Proposed model for the enrichment of transcripts ends: mRNA is isolated (1.), randomly fragmented (2.) and the first 30 nucleotides of the left ends of the fragments are sequenced on an Illumina GenomeAnalyzer (3.). Since the fragmentation of each mRNA produces identical 5′-ends and all other fragments are random, mapping leads to a stack of sequence reads at the mRNA 5′-end (4.) that can be used to annotate the TSS (5.).

To visualize the genome coverage profile of each sample we used two methods for mapping the sequence reads to the positive and negative strands of the PA14 reference genome. The *pileup* profile was generated by counting the number of reads overlapping each genomic position (Figure 1A, upper profile). For the *sinister* profile (introduced by Filiatrault *et al.*, [30]) only the left end (5′-) positions of sequence reads were counted (Figure 1A, lower profile). The method presented here does not produce a uniform coverage of transcripts (Figure 1A). This is probably due to the local RNA secondary structure that may cause preferential and thus non-random fragmentation sites.

The sequence reads of all samples were deposited in the NCBI sequence read archive (SRA) as a study under the accession number SRP009257.

### Quantitative analysis of the *P. aeruginosa* biofilm transcriptome

**Figure 2:**
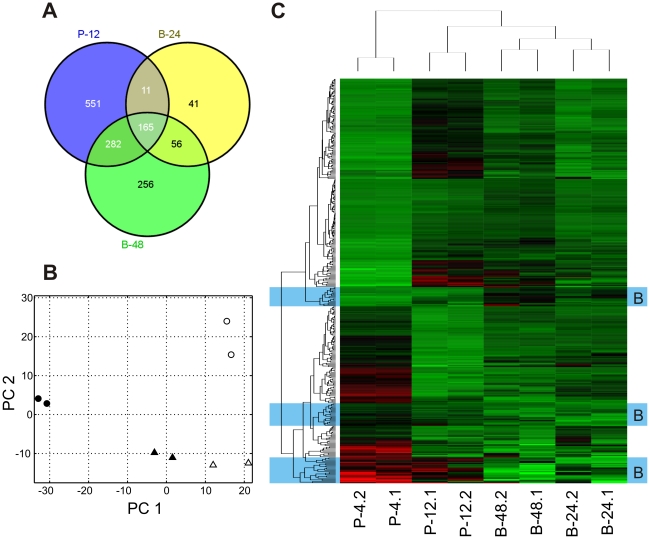
Gene expression under planktonic and biofilm growth conditions. A) Venn diagram showing the genes that were differentially expressed (−2≤log_2_ fold change ≤2; P≤0.001) in planktonic stationary phase cultures (P-12) and biofilms growing for 24 or 48 h (B-24, B-48) as compared to late exponential phase planktonic cultures (P-4). B) Principle component analysis of absolute gene expression. The first principle component (PC 1) accounted for 58% and PC 2 for 25% of the total variation in the dataset. Symbols indicate two biological replicates of late exponential phase planktonic cultures (P-4, •), stationary phase planktonic cultures (P-12, ○), 24 h old biofilms (B-24, ▴) and 48 h old biofilms (B-48, ▵), respectively. C) Cluster analysis of the normalized gene expression for genes that were differentially regulated in P-12, B-24 or B-48 as compared to P-4. Clusters have been labeled ‘B’ for ‘biofilm specific’ (blue background), if gene expression was consistently different between the biofilm cultures (B-24, B-48) and planktonic cultures (P-4, P-12). A comprehensive list of absolute and differential gene expression for all genes included in this figure is provided in Table S2. A larger version of this heatmap including gene labels can be viewed in Figure S1.

In previous studies on global *P. aeruginosa* transcriptional profiles of biofilms, it has been shown that planktonic cultures in exponential and stationary phase of growth and biofilm cultures show distinct patterns of gene expression indicating that biofilms are not just surface attached cells in stationary phase [20], [21]. Similarly, in this study a principal component analysis (PCA) revealed that the biofilm samples from both time points (B-24 & B-48) were clearly distinct from each other as well as from both the late exponential (P-4) and stationary phase (P-12) samples (Figure 2B). In the PCA and a cluster analysis depicted as a heatmap in Figure 2C, the B-24 samples are close to the B-48 samples but show some deviation in the direction of P-4. This can be interpreted as a maturation process of the biofilm with the later B-48 samples representing an older and thus more mature developmental state.

One might have expected a substantial amount of overlap in the expression patterns between stationary phase and static biofilm cultures and the transcriptome of the static biofilm cultures indeed showed a considerable amount of overlap between stationary phase and biofilm cultures (Figure 2A). Many genes that were differentially regulated in B-24 and B-48 compared to P-4 were also differentially regulated in P-12, demonstrating that the static biofilm cultures undergo a similar change in growth conditions towards reduced nutrient and oxygen availability.

Still, the PCA results clearly show that not all of the regulation can be explained by stationary phase adaptions (Figure 2B) and we also found biofilm specific genes that were consistently regulated in all biofilm samples and showed either no or only a much less pronounced differential between P-12 and P-4 (Figure 2C, more detailed results are provided in Figure S1 and Tables S1 and S2).

Among the 682 genes that were up- and 327 genes that were down-regulated in P-12 relative to P-4, many were also differentially expressed in B-24 (227 up- and 46 down-regulated) or B-48 (506 up- and 193 down-regulated). The set of genes that was simultaneously regulated in both stationary phase planktonic and biofilm cultures included genes involved in metabolism and translation (especially ribosomal proteins), which were down-regulated corresponding to a reduced growth rate but also genes involved in flagella (e.g., *flg, fli*) and type IV pili mediated motility (*pil*, *fim*). The quorum sensing dependent virulence factors elastase (*lasA*, *lasB*) and rhamnolipids (*rhlAB*, *rhlC*) [31] were mostly up-regulated in P-12, B-24 and B-48. The differential regulation of those genes likely reflects the combined effect of quorum sensing and nutrient depletion that can be expected to act in a similar way in both stationary phase planktonic batch cultures and static biofilms lacking a continuous in- and outflow of medium.

Among the biofilm specific genes we found three interesting gene clusters. Firstly, the *pel* cluster encoding the biosynthetic genes for the Pel polysaccharide [32], which is an integral part of the EPS matrix, was up-regulated in both B-24 and B-48 but not in P-12. In contrast, the biosynthetic genes involved in the production of the other two major exopolysaccharides associated with *P. aeruginosa* biofilms, Psl and alginate [32], were consistently up-regulated both under biofilm and stationary phase planktonic conditions. Secondly, a large proportion of the biofilm-specific regulation affected genes of the type III secretion system (T3SS), which mediates cytotoxicity by translocating exotoxins to eukaryotic host cells via a needle-like structure that has also been termed the *injectisome* [33], [34]. The genes that were specifically down-regulated in biofilms mostly belonged to the translocation apparatus (*pcrV*, *popBD*) or had a regulatory function (*exs*), while the needle complex itself, encoded by the *psc* genes, was also down-regulated in P-12. Thirdly, a gene cluster that was specifically up-regulated in B-24 and B-48 was *cyoABCD* encoding the *bo_3_* quinol oxidase, one of the five alternative terminal oxidases found in the respiratory chain of *P. aeruginosa*. The *cyo* oxidase is known to be positively regulated by the redox-responsive RoxSR and repressed by the Fur system under iron-rich conditions [35]. Interestingly, the *cbb_3_* type cytochrome c oxidase (*cco*) and the genes for nitrate and nitrite reductase (*nar*, *nir*), which have been described to be favored under microaerophilic or anaerobic growth conditions [36], were down-regulated in the static biofilm cultures.

**Figure 3:**
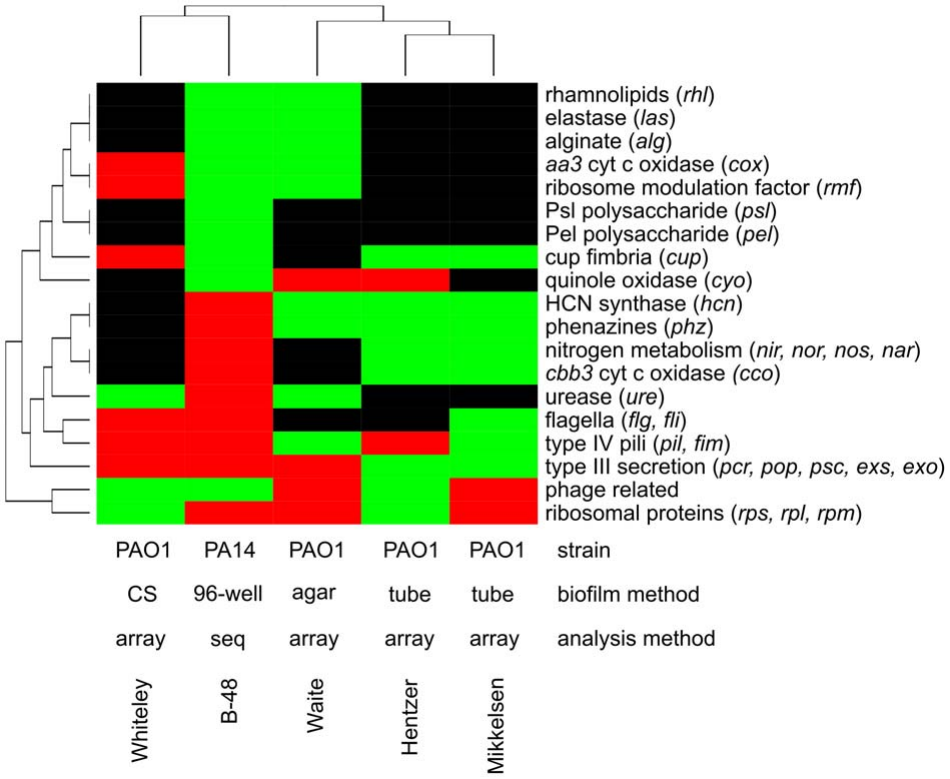
Comparison of differential regulation among different studies. This qualitative ‘heatmap’ compares the differential expression of selected genes and gene clusters in B-48 with similar studies that have been published previously. In all cases, the expression of a biofilm culture was compared to a planktonic culture. The studies included in this analysis are Whiteley *et al.* [1], Waite *et al.* [21], Hentzer *et al.* [4] and Mikkelsen *et al.* [20]. The method abbreviations are CS – chemostat (continuous culture), 96-well – static 96-well plate cultures, agar – nitrocellulose filter on agar surface, tube – silicon tubes, array – DNA microarray analysis, seq – sequencing of cDNA.

A variety of transcriptome studies on gene expression in *P. aeruginosa* biofilms have been published, most of them with the purpose to determine genes that are specifically regulated in developing or mature biofilms and can therefore be assumed to play an important role in the process of biofilm formation [1], [4], [15]–[22]. As depicted in Figure 3 we performed a qualitative clustering analysis and compared the differential expression of selected genes and gene clusters, which have been previously reported to be involved in biofilm formation, across our study and four of the key studies on *P. aeruginosa* biofilm gene expression [1], [4], [20], [21]. Overall, there was only little consistence across the various studies (Figure 3). The highest similarity in gene expression was found between the studies of Hentzer *et al*. [4] and Mikkelsen *et al.* [20]. In those two studies not only the same *P. aeruginosa* strain (PAO1) was used and the data were consistently acquired using microarray technology, but the biofilms were also grown under similar conditions within silicone tubes exposed to a continuous laminar flow.

In our study RNA sequencing was used to determine gene expression in a different *P. aeruginosa* strain background (PA14). Nevertheless, the global gene regulation pattern was not isolated and the relatedness was comparable to that of the four other studies that congruently used microarray technology in the *P. aeruginosa* PA01 background. These results clearly indicate that the environmental conditions of biofilm growth seem to have the most dominant impact on the *P. aeruginosa* transcriptome.

### Expression of small RNAs

The widely used *P. aeruginosa* microarray PaeG1a by Affymetrix (www.affymetrix.com) completely lacks probe sets for small regulatory RNAs (sRNAs), rendering this tool blind towards their expression. However, sRNAs have been assigned central roles in a variety of cellular functions [37], [38] and reports of sRNAs involved in biofilm formation have been published [28], [39]. Several studies have contributed to the detection and annotation of sRNAs in the genome of strain PAO1 [40]–[42], which is the major reference genome of *P. aeruginosa* at the *Pseudomonas* Genome Database (www.pseudomonas.com), but most sRNAs are yet missing in the PA14 genome annotation. Here, we used the list of 31 experimentally confirmed sRNAs published by Sonnleitner *et al.* [37] to identify the orthologous genes in the PA14 genome. With the only exception of PhrD (PA0714.1) all of the 31 sRNAs were identified (Table S3). The differential expression analysis using late exponential phase cultures (P-4) as a reference revealed that 20 sRNAs were up-regulated in stationary phase or biofilm cultures, 3 were down-regulated and 1 (PhrS) was inversely regulated showing up-regulation in P-12 and down-regulation in B-48 (Table 1). Similar to the global pattern, many sRNAs were up-regulated simultaneously in P-12 and in the biofilm cultures including RsmY, RsmZ, RgsA, PrrF1/2 and CrcZ. The two sRNAs RsmY and RsmZ, belong to a regulatory pathway that is activated by the two-component system GacS/GacA [28], [43], which also controls the sRNA RgsA [42]. RsmY and RsmZ antagonize the translational regulator RsmA. The whole regulatory cascade involving RsmY/Z, RsmA and GacS/A is additionally controlled by at least two other sensor kinases RetS and LadS [37], [43]. Of note, the latter, LadS, which has been shown to be an activator of biofilm formation in *P. aeruginosa* PAK [28], has no functional homolog in PA14. Several sRNAs showed a biofilm-specific expression pattern. These included the 4.5 S and 6 S RNA that were highly up-regulated in biofilms while their expression was only moderately increased in P-12 (Table 1). 4.5 S RNA is encoded by the *ffs* gene and is a part of the bacterial signal recognition particle (SRP), which promotes the recognition and insertion of integral membrane proteins [44] and which has been shown to be required for virulence and biofilm formation in *Streptococcus* [45], [46]. The other, 6 S RNA, is known to transcriptionally repress the expression of σ^70^ dependent genes in *E. coli* by binding the σ^70^-RNA-polymerase complex [47]. The sRNA PhrS was inversely regulated in P-12 (up) and B-48 (down). It has recently been shown that PhrS stimulates the synthesis of the 4-quinolones by direct interaction with the mRNA of the transcriptional regulator PqsR [48].

**Table 1:** Small RNAs that were differentially expressed in PA14 biofilms.

	PA14 locus			log_2_ fold change^b^		
gene name^a^	start	end	strand	P-12	B-24	B-48
**upregulated in biofilms**						
P1	334549	334728	-	1.48		
RsmY	586867	586990	+	5.15	2.41	5.81
P9	1436491	1436618	-	1.59		
4.5 S RNA (*ffs*)	1668911	1669081	-	2.56	5.01	5.23
sRNA1059	1996807	1997508	?	4.47		4.14
sRNA1466	2918212	2918965	?			2.35
RgsA	3318747	3318868	-	5.80	3.21	10.44
sRNA1714	3360654	3360873	-	1.79		3.20
amiE leader	3778034	3778133	+	3.65		3.00
RsmZ	4057543	4057658	+	5.21	2.23	5.89
sRNA2315	4536493	4536919	+			1.38
sRNA2626	5196833	5197184	+		3.21	2.32
PrrH	5283995	5284319	+	12.08	6.50	10.37
PrrF1	5283995	5284110	+	8.83	5.21	7.04
PrrF2	5284207	5284319	+	12.45	6.62	10.58
crcZ	5308587	5308993	+	3.56		2.87
P30	5308743	5308964	-			4.88
P32	5344950	5345060	-	1.25		2.23
P34	5835082	5835480	-	1.94		3.16
6 S RNA (*ssrS*)	5884320	5884502	+	2.36	4.48	4.85
**downregulated in biofilms**						
sRNA622	1140860	1141267	-		−2.54	−3.65
PhrS	3705309	3705521	+	1.28		−1.74
72/101	4939194	4939277	-			−2.14
P26	4780768	4780833	+	−1.23		

a : annotation taken from Sonnleitner et al.

b : relative to P-4, only at least +/− 1 log2-fold regulated genes with a P-value < = 0.001 are shown.

### Qualitative analysis of the *P. aeruginosa* transcriptome

An additional important advantage of using RNA-seq instead of array-based methods for transcriptional profiling is the inherent possibility to gain not only quantitative but also qualitative data about the transcriptome. Since the method we used to generate cDNA libraries is based on the ligation of adapters to both ends of randomly fragmented RNAs, the sequencing data should cover the full length of transcripts that are actually present in the sample. The visualization of the genome coverage profile (Figure 1) shows a characteristic enrichment of sequence reads in promoter regions turning up as single peaks in the *sinister* profile or as elevated coverage over the length of a single read in the *pileup* (Figure 1A). Since in most cases these peaks also marked the beginning of a contiguous stretch of read coverage spanning a whole gene or operon, it seemed likely that they represent transcriptional start sites (TSS). The observed enrichment of transcript left ends that represent TSS seems to be a method specific phenomenon that can be exploited for a qualitative analysis of the transcriptome. Figure 1B presents a model of how the enrichment of TSS may be explained. In principle, the RNA fragmentation produces random left ends that are subsequently sequenced resulting in principle in a random coverage of the transcripts. However, the 5′-end of a transcript is always present in the leftmost fragment, independent from the fragmentation points, which explains the overrepresentation of reads that originate from transcript left ends.

### Multiple transcriptional starts sites of the *pqsABCDE* operon

**Figure 4:**
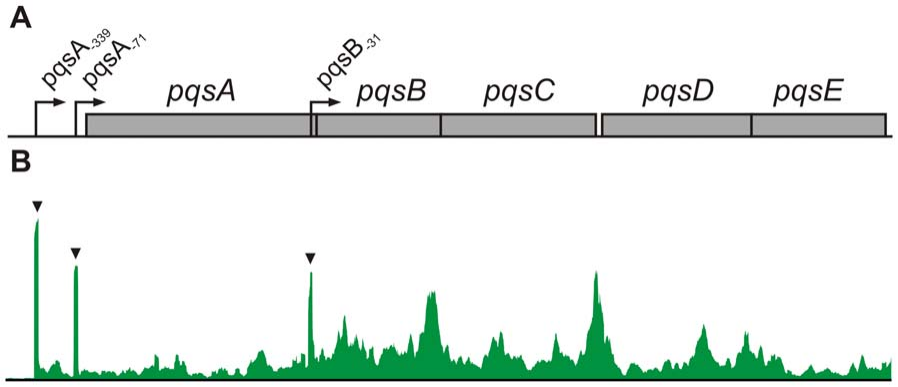
Alternative transcriptional start sites of the PQS operon. A) Model of the PQS biosynthetic operon (*pqsABCDE*) with the three alternative TSS indicated by arrows. The TSS are named by the next downstream gene with subscripts indicating the TSS position relative to the start of the associated gene. B) *Pileup* profile of the PQS operon depicting the total amount of read counts generated in this study that map to the PQS operon. The enriched TSS loci are indicated by black triangles.

The PQS-operon (*pqsABCDE*, Figure 4A) encodes genes that are essential for the biosynthesis of hydroxy-alkylquinolones (HAQs) including 3,4-dihydroxy-2-heptylquinolone also known as the Pseudomonas Quinolone Signal (PQS) [49], [50]. So far, only one TSS was described for the PQS-operon placed 71 bp upstream of the *pqsA* start codon [51]. Our RNA-seq analysis detected this canonical TSS and additionally revealed a secondary TSS placed 339 bp upstream from the *pqsA* start and surprisingly also a third TSS placed within the coding sequence of *pqsA* and 31 bp upstream of the start codon of the second gene in the operon, *pqsB* (Figure 4B). In order to confirm that the observed peaks actually represent TSS, we sequenced the 5′-ends of the mRNAs by rapid amplification of cDNA ends (5′-RACE). Briefly, the specific mRNAs were reverse transcribed and the resulting cDNAs were poly-adenylated on their 3′-ends. An anchor sequence was introduced into this poly-A-tail by PCR amplification that subsequently enabled the sequencing of the product by Sanger technology and the identification of the start site of the original mRNA. The *pqsA* and *pqsB* mRNAs and two additional (*mexR* and *mexA*) mRNAs that were tested contained five individual putative TSS, all of which were confirmed to be located at the position that has been predicted by the global transcriptome sequencing (Table 2).

**Table 2:** Confirmation of predicted transcriptional start sites (TSS) by 5′-RACE.

peak	Gene^a^	PA14 no^a^	sequence context^b^	TSS locus		distance to next gene
				5′-RACE^c^	RNA-seq	
pqsA_−71_	*pqsA*	PA14_51430	GTTCC[TG]ACAAAGCAAG	4571540-41	4571540	71
pqsA_−339_	*pqsA*	PA14_51430	ATTGG[TTTG]CCATCTCATG	4571808-11	4571808	339
pqsB_−31_	*pqsB*	PA14_51420	CACCC[TTTA]TCACGACAAC	4569950-53	4569953	31
mexR_−41_	*mexR*	PA14_05520	TTGAC[TG]GATCAACCAC	486540-41	486541/44^d^	41/44^d^
mexA_−21_	*mexA*	PA14_05530	GCCGC[TTTC]GCTCATGAGG	486753-56	486754	21

a : Annotation according to www.pseudomonas.com.

b : TSS-location as predicted by RNA-seq is underlined, possible TSS locations as detected by 5′-RACE are embraced in square brackets. The ambiguity results from poly-A tailing (see text).

c : Numbers correspond to the position range of the putative TSS nucleotides as marked by square brackets in the sequence context.

d : RNA-seq data showed two adjacent peaks upstream of *mexR*. The smaller one (−44) was not confirmed by 5′-RACE, probably due to its low abundance.

The canonical TSS *pqsA*_−71_ is associated with a putative −10-box (TAGTTT) of the housekeeping sigma factor RpoD (σ^70^) and a binding site for the PQS regulatory protein MvfR (also named PqsR) [51]. It is therefore likely that the TSS *pqsA*_−71_ represents the ‘main’ start of the 4-quinolone regulated *pqsABCDE* transcription. The second TSS *pqsA*_−339_ does not contain any characterized −10 motif but an RhlR-binding site, known to inhibit expression of *pqsABCDE* [51], is situated at the corresponding −35 position. The third TSS located upstream of *pqsB* was unexpected. The expression of a transcript containing only *pqsBCDE* seems counterintuitive, as PqsA is an anthranilate-coenzyme A ligase that provides anthraniloyl-CoA, which is essential for the synthesis of HAQs [52]. Nevertheless, all three TSS were independently confirmed by 5′-RACE (Table 2).

The presence of multiple TSS for one operon clearly adds another level of complexity to the regulation of gene expression. For a marine bacterium of the *Pseudoalteromonas* genus it was recently reported that its chitinase operon consisting of three genes *chiABC* is expressed using as many as 13 different TSS that are differentially used depending on the nutrient regime [53]. This way gene expression can not only be directly influenced by various transcription factors and/or alternative sigma factors but the expression of transcripts with untranslated regions (UTRs) at the 5′-end of various lengths may significantly impact on posttranscriptional regulation.

### Genome-wide detection of transcriptional start sites

**Figure 5:**
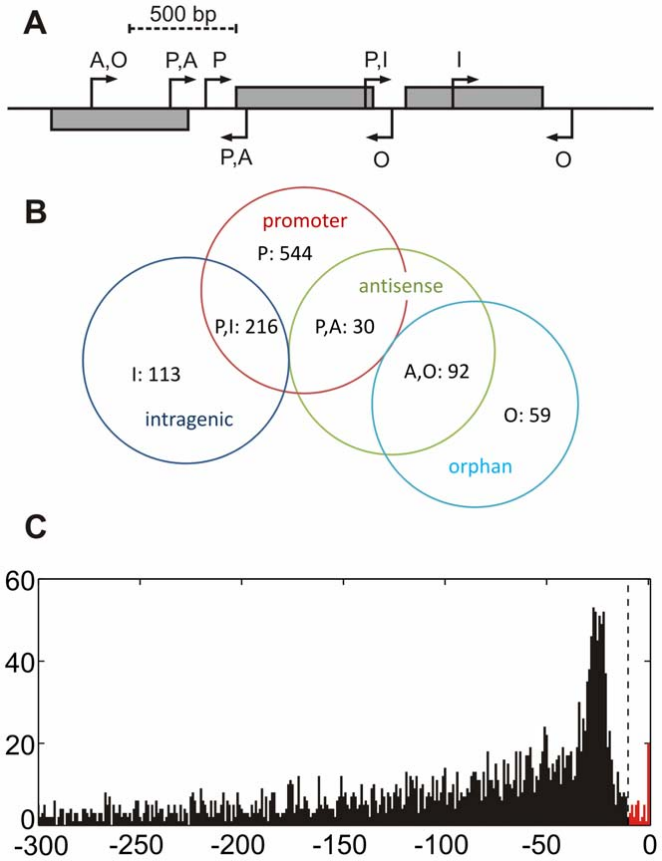
Detection of transcriptional start sites. A) Transcriptional start sites (TSS) were categorized by their position relative to annotated genes as follows: promoter (P), up to 500 bp upstream of an annotated gene on the same strand, intragenic (I), within an annotated gene on the same strand, antisense (A), within an annotated gene on the opposite strand, orphan (O), neither ‘intragenic’ nor ‘promoter’, which means no association to any annotated gene. B) Venn diagram depicting the abundance of the different categories among 1024 TSS that were independently detected for at least two different conditions. C) Distribution of TSS in promoter regions of coding genes (class ‘P’), indicating the length of the untranslated regions (UTRs) at the 5′ end of mRNAs. The peak of the distribution is located around the −25 position. The dashed line marks the −10 nt position, which indicates the 43 leaderless transcripts (red bars) with 5′ UTRs of less than 10 nt length.

The characteristic enrichment of putative TSS enabled us to screen the global *sinister* profiles of our eight samples (two replicates of each of the four culture conditions, P-4, P-12, B-24 and B-48) for putative TSS sites by looking for an increased absolute and relative peak height (as compared to the local *sinister* profile) and for *local transcript enrichment* (the increase in read counts following a TSS). To increase specificity of this detection method only those peaks were accepted as putative TSS, which were positively detected in both replicates of one of the four culture conditions (Table S4). This resulted in the detection of 3389 putative transcriptional start sites including 1054 that were found in at least 2 culture conditions. As it was already shown for other bacterial organisms [30], [54], many promoter regions in *P. aeruginosa* contained multiple TSS and TSS were also found abundantly within predicted operons or in antisense orientation to annotated genes. Therefore, we categorized the putative TSS into four classes (Figure 5A): firstly, those that are located within promoter regions (P) of annotated genes (within a 500 bp sequence upstream of the translation start site), secondly, intragenic TSS that localize within an annotated gene (I) on the same strand; thirdly, antisense TSS that are ‘intragenic’ but on the opposite strand (A), and fourthly, orphan TSS that locate neither in promoter regions nor inside annotated genes on the same strand (O). By definition, the categories are partially overlapping and TSS can be in a promoter region that overlaps a gene that is located upstream on the same strand (P,I) or opposite strand (P,A) and orphan TSS may be located antisense to an annotated gene (A,O). The Venn diagram shown in Figure 5B provides an overview of the classification of the 1054 TSS loci that were found in at least two conditions. The majority (75%) of these putative TSS are located in promoter regions. The position of the promoter TSS relative to the associated downstream gene exhibits a peak around the −25 bp position (Figure 5C). This distribution of the lengths of 5′ untranslated regions (5′-UTRs) is highly similar to the distribution that has been described for *H. pylori* [54]. Figure 5C also reveals the presence of 43 mRNAs with very short (less than 10 nt) 5′-UTRs that are likely to represent leaderless transcripts, which lack a Shine-Dalgarno site for translation initiation. Thus, like in other γ-Proteobacteria [55], the amount of leaderless mRNAs appears to be low in *P. aeruginosa*.

The detection algorithm also identified 405 antisense TSS, 122 of which were found in at least two of the four culture conditions (Figure 5B, Table S4). The majority are not associated with overlapping promoters on opposite strands (92 A,O sites, Figure 5B) and might be potential start sites for un-annotated antisense transcripts. In addition, the global screening also identified 543 orphan TSS (151 in at least two conditions) that were not associated with any annotated gene (class O, Figure 5B). These results demonstrate the possibility to detect previously un-annotated genes by the use of RNA sequencing technology.

Nevertheless, in order to provide robust data on the genome-wide detection of TSS, operon structures, the presence of novel sRNAs and previously un-annotated genes, not only a significant improvement in the qualitative and quantitative content of the sequencing data, e.g., by producing longer sequence reads and paired end sequencing to cover also the 3′-ends of fragments, will be an absolute requirement. But for the re-annotation of the *P. aeruginosa* genome also a plethora of RNA sequence data generated from *P. aeruginosa* cultured under diverse environmental conditions are needed in order to enforce a truly genome-wide map of transcripts.

### Conclusions

The aims of this study on gene expression in *P. aeruginosa* biofilms were twofold: we wanted to firstly determine the genes that are specifically regulated under biofilm conditions in the static 96-well plate system that was recently introduced by Müsken *et al.* [23], [24] and secondly, to gain additional information about the qualitative transcriptome.

By using RNA-seq we were able to analyze the PA14 transcriptome to its full extent including the accessory genomic elements and small regulatory RNAs that are missing from the widely used PaeG1a microarray by Affymetrix, which is based on the genome of strain PAO1. The comparative analysis of gene expression in planktonic and biofilm cultures revealed genes that were simultaneously regulated in the stationary phase of planktonic cultures and in biofilms as well as a set of genes that were specifically regulated in biofilms. The latter included a large proportion of genes previously described to be biofilm regulated such as genes involved in type three secretion, adaptation to microaerophilic growth and the production of extracellular matrix components.

Furthermore, a qualitative analysis of the RNA-seq data revealed that the 5′-ends of the original transcripts were enriched in the sequence data enabling an accurate prediction of transcriptional start sites (TSS). Such qualitative information on the transcriptome adds a new layer of complexity to the analysis of gene expression that can be exploited without the application of additional methods. As an important example, we have identified three alternative TSS of the biosynthetic operon *pqsABCDE*, which is essential for the synthesis of the *Pseudomonas* quinolone signal PQS, a key player in the global control of virulence factor production. Future work will have to shed light on the biological meaning of these multiple TSS and their individual importance for the regulation of the expression of interbacterial 4-quinolone signaling molecules.

RNA seq has several additional advantages. Because of the use of barcoded cDNA libraries, the method is easily scalable allowing adjusting cost and data output according to the scientific and economic requirements. Deeper sequencing will further decrease the detection limit for lowly expressed genes and thereby increase the dynamic range of gene expression that can be analyzed. Furthermore, using longer sequence reads or paired end sequencing (the sequencing of both ends of the cDNA fragments) will produce larger overlaps in the read data that enable an accurate prediction of sequence variations and also the *de novo* assembly of the transcriptome. This will be particularly interesting in the analysis of strains that have not (yet) been fully sequenced like clinical or environmental isolates.

## Methods

### Bacterial strains and growth conditions

*Pseudomonas aeruginosa* PA14 was used for all experiments. RNA was prepared from exponentially growing (P-4) and stationary phase (P-12) planktonic cultures and biofilms after 24 h (B-24) and 48 h (B-48). Two biological replicates of each condition were prepared and analyzed in this study. The following protocol describes the procedure for one single sample.

For exponentially growing planktonic cultures, 3 independent pre-cultures were incubated overnight in glass tubes filled with 4 mL LB at 37°C, shaking at 180 rpm. A 10 mL main culture was prepared from each pre-culture in 10 mL fresh LB starting with an OD_600 nm_ of 0.02 and incubated at 37°C, shaking at 180 rpm. Cultures were pooled after reaching an OD_600 nm_ of 2.0 +/− 0.1 (approximately 4–5 h after incubation, P-4) or after 12 h (P-12) and mixed with an equal volume of RNA-Protect buffer (Qiagen). After 10 min incubation at room temperature, aliquots of 1 mL were centrifuged at 6000×g for 5 min, the supernatant was removed and the pellets were stored at −70°C.

For biofilm cultures, 3 independent pre-cultures were incubated overnight in glass tubes filled with 4 mL LB at 37°C, shaking at 180 rpm. From each pre-culture, 8 wells of a 96-well μClear microplate (Greiner Bio-One) containing 100 µL LB per well were inoculated by diluting the pre-culture to an OD_600 nm_ of 0.02. The biofilm plates were sealed with an air-permeable cover foil (Greiner Bio-One) and placed in a humid incubator at 37°C. After 24 h (B-24) or 48 h (B-48), respectively, all 8 wells were pooled and subsequently harvested as described above for the planktonic cultures.

### Design of adapter oligonucleotides and barcodes

The RNA adapters that were ligated to the fragmented RNA were designed for compatibility with Illumina GenomeAnalyzer flow cells to allow sequencing of the final cDNA libraries without any further manipulations. A full list of all oligonucleotides is presented in Table S5. All oligonucleotides were purchased from Eurofins MWG Operon. The 3′-adapters were synthesized with a 5′-phosphate residue (P) to enable ligation to 3′-ends of the RNA fragments and a 3′-dideoxycytidine (ddC) to prevent the polymerization of primers. Barcode sequences of 6 nt length were included at the 3′-ends of 5′-adapters, adjacent to the binding site of the Illumina sequencing adapter. Thus, any fragment that is correctly sequenced should produce reads starting with the barcode sequence followed by the original transcript sequence. The barcodes were designed to differ at least at three positions to prevent false detections caused by sequencing errors.

### Preparation and sequencing of cDNA libraries

RNA was extracted from cell pellets using the RNeasy Mini Kit (Qiagen) in combination with Qiashredder columns (Qiagen) according to the manufacturer's instruction with some modifications. To deplete ribosomal RNA sequences, the samples of isolated total RNA were treated with the MICROBExpress Bacterial RNA enrichment kit with the *Pseudomonas* module (Ambion) according to the manufacturer's instructions. The RNA was fragmented by sonication using a Covaris Adaptive Focused Acoustics device (Covaris) to a median fragment size of ∼200 nt, purified using RNeasy MiniElute columns (Qiagen) and checked for quality and size distribution on a Bioanalyzer Pico Chip (Agilent). RNA fragments were 5′-phosphorylated using T4 polynucleotide kinase (Fermentas) and consecutively ligated to 3′- and 5′-RNA-adapter oligonucleotides (Table S5) using T4 RNA ligase (Fermentas). The resulting RNA libraries were reverse transcribed using SuperScript II (Invitrogen) together with a DNA primer complementary to the 3′-adapter sequence to generate first strand cDNA (Table S5). To enrich for correctly ligated and reverse transcribed products, cDNA libraries were amplified by 15 cycles of polymerase chain reaction using the *Pfu* polymerase (Promega) with PCR primers identical to corresponding Illumina primers to ensure full compatibility with GenomeAnalyzer flowcells (Table S5). Prior to sequencing, the final cDNA libraries were additionally normalized using double strand specific nuclease (Evrogen) as described in the “DSN Normalization Sample Preparation Guide” by Illumina for additional depletion of ribosomal cDNAs.

The final cDNA libraries were ready for sequencing on an Illumina Genome Analyzer II_x_ without further treatment. Barcoded 5′-adapters enabled the pooling of multiple samples on one lane of the Illumina flow cell. Libraries were sequenced with 36 cycles in single end mode, which resulted in 36 nt long reads including 6 nt of barcode and 30 nt of the sequenced 5′-ends of fragments.

Cluster generation was performed using the Illumina cluster station. Sequencing for cDNA fragments on the Genome Analyzer followed a standard protocol. The fluorescent images were processed to sequences using the Genome Analyzer Pipeline Analysis software 1.8 (Illumina).

### Computational analysis

Raw sequence data obtained in Illumina FASTQ-format were first separated by their barcode sequence by comparing the first 6 bases with the expected barcode sequences. Successfully detected barcodes were removed from the sequence leaving reads of 30 nt length, while reads containing no recognizable barcode sequence were discarded. Reads with a barcode sequence differing at only one position were also included in the analysis to allow for single sequencing errors and to increase data output. The reads were mapped to the PA14 reference genome, which is available for download from the *Pseudomonas* Genome database (http://v2.pseudomonas.com, [56]). Mapping was performed using *bowtie* (http://bowtie-bio.sourceforge.net, [57]) with options ‘-m 1’, ‘–best’ and ‘–strata’ to allow only uniquely mapping hits and to avoid problems with repeat regions and remaining ribosomal reads (the PA14 contains 4 nearly identical rRNA gene clusters). For the purpose of visualization of transcript coverage and quantitative analysis of differential gene expression, two similar types of profiles were calculated from the mapped sequence reads. The *pileup* profile was calculated by counting the number of reads that overlapped with a specific position independently for each strand:
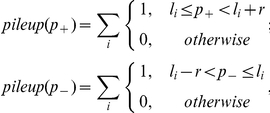
where *p_+_* and *p_−_* are positions on the plus and minus strand, respectively, *l_i_* is the mapped position of the left (5′-) end of sequence read *i* and *r* is the read length (without barcode, here: 30 nt). The *sinister* profile (see also Filiatrault *et al.*, [30]) was similarly calculated by counting only the 5′-ends of each read:
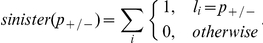



The *Artemis* genome browser was used for visualization of the *pileup* and *sinister* profiles (http://www.sanger.ac.uk/resources/software/artemis/, [58]) as depicted for example in Figure 1A.

For the quantitative analysis of gene expression, the read counts per gene (RPG), were calculated for each annotated gene by summing up the *sinister* profile overlapping with the gene locus:
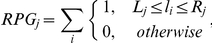
where *L_j_* and *R_j_* are the left and right margins of gene *j* and *l_i_* is the 5′-end location of read *i*. Only reads that mapped to the same strand were included in the calculation.

The RPG values of all genes were calculated for each condition independently and put into a matrix that was subsequently analyzed using DESeq for differential expression analysis, a software package for R that uses a statistical model based on the negative binomial distribution [59].

A principle component analysis (PCA) was performed on normalized read counts of all eight samples to compare gene expression under the four different conditions. First, read counts were normalized by the gene length to obtain RPK values (Reads Per Kilobase):
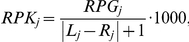
where *L_j_* and *R_j_* are the left and right margins of gene *j*. Because genes with low absolute read counts show a higher variation due to background noise, only 842 genes that showed RPK values of at least 32 in all eight samples were included in the PCA. The RPK where then divided by a size factor that is a measure for the total amount of reads of the sample. The size factor method was adapted from DESeq and was used instead of the widely used RPKM normalization [60], because it is more robust towards differences in the abundance of highly expression genes. Size factors were calculated using the following equation:
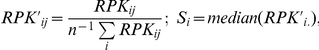
where *RPK_ij_* is the RPK value of gene *j* in sample *i* and Σ*_i_ RPK_ij_* is the sum of RPK values of gene *j* throughout all *n* samples. In other words, for each gene, the RPK value is divided by the mean of RPK values of the same gene over all samples and the size factor *S_i_* is the median of these normalized RPK values for sample *i*. The RPK values where divided by the size factors to yield normalized read counts that are comparable among all genes and all samples:
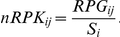



Finally, the PCA was performed on the base 2 logarithms of normalized RPK values using the ‘princomp’ function of the statistics toolbox in MATLAB R2007b (The MathWorks).

A cluster analysis of the *nRPK* values was performed in R for all genes that were differentially expressed (i.e. abs(log_2_ fold change) ≥2 & adjusted P-value≤0.001) in P-12, B-24 or B-48 as compared to P-4, showed no infinite fold changes (which occur, if the read count of a gene is zero) and which were annotated with a gene name (i.e., in most cases, a four-letter label like *dnaA*). A distance matrix was calculated using the *dist* function and a hierarchical clustering was performed using *hclust*. Individual clusters were identified using *cutree* with k = 20. The heatmap corresponding to the hierarchical clustering was generated with the *heatmap.2* function of the R package *gplots* (http://cran.r-project.org/web/packages/gplots/index.html).

### Identification of transcriptional start sites (TSS)

In order to detect putative transcriptional start sites, the *sinister* profiles of all 8 samples were analyzed individually. Two values were calculated at each position of both strands: i) *local relative peak height*, the ratio of the absolute *sinister* value divided by the number of 5′ read ends in a window of 120 nt centered on the position ( = 2 times the readlength up- and downstream) and ii) *local transcript enrichment*, the ratio of number of 5′ read ends in a window reaching 60 nt downstream divided by the number of 5′ read ends in a window reaching 60 nt upstream. A TSS was called, if the following three criteria were fulfilled for both replicates of at least one of the four conditions (P4, P12, B24, B48) at its genomic position: i) *absolute peak height* (i.e., the *sinister* profile) ≥5, ii) *local relative peak height* ≥0.25 and iii) *local transcript enrichment* ≥2.

### Sequencing of mRNA 5′-ends (5′-RACE)

Five transcriptional start sites were additionally confirmed by rapid amplification of 5′ complementary DNA ends (5′ RACE) by a protocol that was modified from [61]. Briefly, purified RNA was reverse transcribed using a primer specific for the target gene (see Table S5 for primer sequences). The cDNA was treated with Terminal deoxynucleotide Transferase (Roche) in the presence of dATP to polyadenylate 3′-end of the cDNA. Subsequently, the target cDNA was PCR amplified by using a three primer mix: a linker primer containing a 3′-poly-dT sequence and an 5′-anchor sequence, a second primer containing only the anchor sequence and a gene specific primer targeting a sequence downstream of the gene specific primer that was used for reverse transcription to increase specificity in a ‘half-nested’ PCR reaction. PCR products were sequenced by the Sanger method on a 3730×l DNA Analyzer (Applied Biosystems).

### Annotation of small RNAs

For a detailed analysis of the expression of sRNAs we extracted the genomic sequences of all small RNAs listed for PAO1 in Sonnleitner *et al.* [37] and performed a BLASTN search against the PA14 genome. The hits showing more than 90% sequence similarity and full-length coverage were manually added to the PA14 annotation. For the PhrD gene no homolog was found in the PA14 genome. All other sRNAs listed by Sonnleitner *et al.* were found and annotated. Additionally, we identified the small RNA P36, which is annotated as PA4726.1 but missing from the Sonnleitner *et al.* collection of sRNAs.

## Supporting Information

Figure S1 **Cluster analysis of gene expression in planktonic and biofilm cultures.** This figure provides a larger version of Figure 2C. In addition, the gene annotation is displayed on the right margin including the locus ID for PA14 according to the annotation in the *Pseudomonas* genome database [56] and the gene name. The small RNA genes that have been identified in this study and are not included in the PA14 annotation have been assigned the locus ID of their PAO1 ortholog in accordance with Table S3. Horizontal lines separate the 20 clusters (numbers indicated on the left margin) of the underlying hierarchical clustering, from which the 3 biofilm specific clusters highlighted with a blue background color were derived.
(TIF)

Table S1 **Genes that were differentially regulated in P-12, B-24 or B-48 as compared to P-4.**
(XLS)

Table S2 **Cluster analysis of genes that were differentially expressed in P-12, B-24 or B-48.**
(XLS)

Table S3 **Identification of sRNAs in the** ***P. aeruginosa*** **PA14 genome.**
(XLS)

Table S4 **Putative transcriptional start sites (TSS).**
(XLS)

Table S5 **List of RNA and DNA oligonucleotides used in this study.**
(XLS)
