# Supplementary figures and images for: The Pseudomonas aeruginosa Transcriptome in Planktonic Cultures and Static Biofilms Using RNA Sequencing
Source: PLoS One. 2012 Feb 3;7(2):e31092. doi: 10.1371/journal.pone.0031092 (PMC3272035; doi:10.1371/journal.pone.0031092)

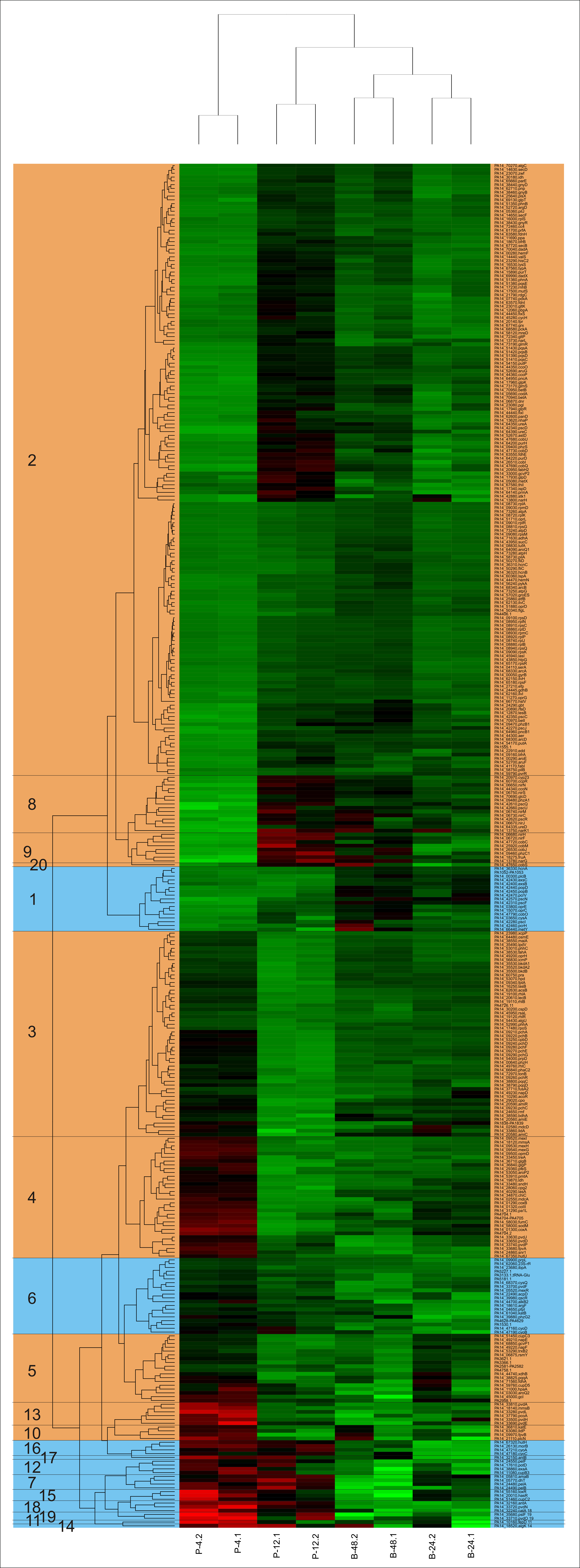

Supplement: Figure S1 — Cluster analysis of gene expression in planktonic and biofilm cultures. This figure provides a larger version of Figure 2C. In addition, the gene annotation is displayed on the right margin including the locus ID for PA14 according to the annotation in the Pseudomonas genome database [56] and the gene name. The small RNA genes that have been identified in this study and are not included in the PA14 annotation have been assigned the locus ID of their PAO1 ortholog in accordance with Table S3. Horizontal lines separate the 20 clusters (numbers indicated on the left margin) of the underlying hierarchical clustering, from which the 3 biofilm specific clusters highlighted with a blue background color were derived. (TIF) [file pone.0031092.s001.tif]
